# Fluorogenic RNA Aptamers: A Nano-platform for Fabrication of Simple and Combinatorial Logic Gates

**DOI:** 10.3390/nano8120984

**Published:** 2018-11-28

**Authors:** Victoria Goldsworthy, Geneva LaForce, Seth Abels, Emil F. Khisamutdinov

**Affiliations:** Department of Chemistry, Ball State University, Muncie, IN 47304, USA; vgoldsworthy@alex.k12.in.us (V.G.); grlaforce@bsu.edu (G.L.); sethabels1982@gmail.com (S.A.)

**Keywords:** logic gates, nucleic acid computing, RNA aptamers, RNA nanotechnology

## Abstract

RNA aptamers that bind non-fluorescent dyes and activate their fluorescence are highly sensitive, nonperturbing, and convenient probes in the field of synthetic biology. These RNA molecules, referred to as light-up aptamers, operate as molecular nanoswitches that alter folding and fluorescence function in response to ligand binding, which is important in biosensing and molecular computing. Herein, we demonstrate a conceptually new generation of smart RNA nano-devices based on malachite green (MG)-binding RNA aptamer, which fluorescence output controlled by addition of short DNA oligonucleotides inputs. Four types of RNA switches possessing AND, OR, NAND, and NOR Boolean logic functions were created in modular form, allowing MG dye binding affinity to be changed by altering 3D conformation of the RNA aptamer. It is essential to develop higher-level logic circuits for the production of multi-task nanodevices for data processing, typically requiring combinatorial logic gates. Therefore, we further designed and synthetized higher-level half adder logic circuit by “in parallel” integration of two logic gates XOR and AND within a single RNA nanoparticle. The design utilizes fluorescence emissions from two different RNA aptamers: MG-binding RNA aptamer (AND gate) and Broccoli RNA aptamer that binds DFHBI dye (XOR gate). All computationally designed RNA devices were synthesized and experimentally tested in vitro. The ability to design smart nanodevices based on RNA binding aptamers offers a new route to engineer “label-free” ligand-sensing regulatory circuits, nucleic acid detection systems, and gene control elements.

## 1. Introduction

The progression in the field of RNA nanotechnology makes RNA molecules the most promising candidate to fabricate bio-computers due to their variable folding properties as well as their catalytic functions [1,2]. Numerous non-canonical nucleotide interactions, found only in RNA [3,4], enable this biopolymer to self-assemble into various shapes and dimensions as exemplified by naturally occurring ribosomal RNA [5] and ribozymes [6,7] as well as by artificially constructed RNA polygons [8,9,10,11,12], prisms, and cubes [13,14,15]. This diverse structural capability of RNA led to the development of aptamer technology almost 30 years ago [16,17]. Aptamers are single-stranded RNA or DNA oligonucleotides, with typical length of no more than 100 nts that were artificially selected from combinatorial libraries for high binding affinities to specific molecular targets. Since their development, aptamers have revolutionized the field of biosensing by enabling scientists to rationally generate different aptamers targeting a diverse range of ligands [18,19,20]. RNA-based fluorogenic modules are of particular interest [21,22,23] since they have applications in monitoring gene expression [24,25] and new drug screening pipelines using microarrays developed to sense target molecules of variable size [26]. This florescence module includes a light-up RNA aptamer and fluorogen. The light-up RNA aptamers are selected to specifically bind to small organic molecules exhibiting minimal to no fluorescent emission when free in solution (fluorogen or fluorogenic dyes) and trigger its florescence. The well-studied examples include malachite green (MG)-binding RNA aptamer [27], and 3,5-difluoro-4-hydroxybenzylidene imidazolinone (DFHBI)-binding RNA aptamers [28,29] among others [30,31].

In RNA nanotechnology, the development and implementation of RNA-based nanodevices that respond to biomolecular inputs by generating output signals in accordance with logic gate behavior has attracted considerable attention [32,33,34,35,36]. Computing using both RNA and DNA molecules integrates biochemistry and molecular biology disciplines to achieve a certain goal through designing algorithmic processes embedded within polynucleotide structures. The concept of using nucleic acids (NAs) for computation was proven in 1994 when Leonard Adleman demonstrated the ability of synthetic DNA oligonucleotides to solve a seven-point Hamiltonian path problem [37] and, since then, many studies have reviewed the possibility of developing a new generation of molecular logic gates and molecular computers based on nucleic acids [38,39]. In contrast to silicon-based computers, NA computers implement concentrations of specific molecules, such as metal ions, small organic dyes, single stranded DNA or RNA oligonucleotides, peptides or proteins, as inputs to derive certain signals, e.g., switching between RNA conformations, activation or deactivation of ribozyme activity, down- or up-regulation of certain genes, etc. [40,41,42,43,44,45]. This relies on the algorithmic processes carefully designed and encompassed within a nucleic acid complex (referred to as logic gates (LGs)) that are capable of performing simple AND, OR, NAND, and NOR logic operations, as well as more sophisticated logic circuits.

DNA has been routinely used for the development of biochemical circuits and all basic logic operations, including INHIBIT, IMPLICATION, and XOR have been mimicked with DNA as a template [46,47,48,49,50]. There are also various classes of functional RNA molecules, such as ribozymes, riboswitches, miRNA, siRNA, and orthogonal ribosomes, that enable the fabrication of computational systems [51,52,53,54] and simple RNA fluorogenic biosensors [55,56]. However, it is often essential to develop higher-level logic circuits for the production of multi-task nanodevices for data processing, which usually require combinatorial logic gates [57,58]. For example, a half adder can perform an addition operation on two binary digits by integration of an XOR gate and an AND gate in parallel to generate a SUM (S) output and a CARRY (C) output, respectively. To the best of our knowledge, the development of combinatorial RNA logic gates based on light-up RNA aptamer fluorogenic systems has yet to be realized, and would represent a label-free oligonucleotide bio-sensing platform with potential applications in biocomputing and biosensing.

**Figure 1:**
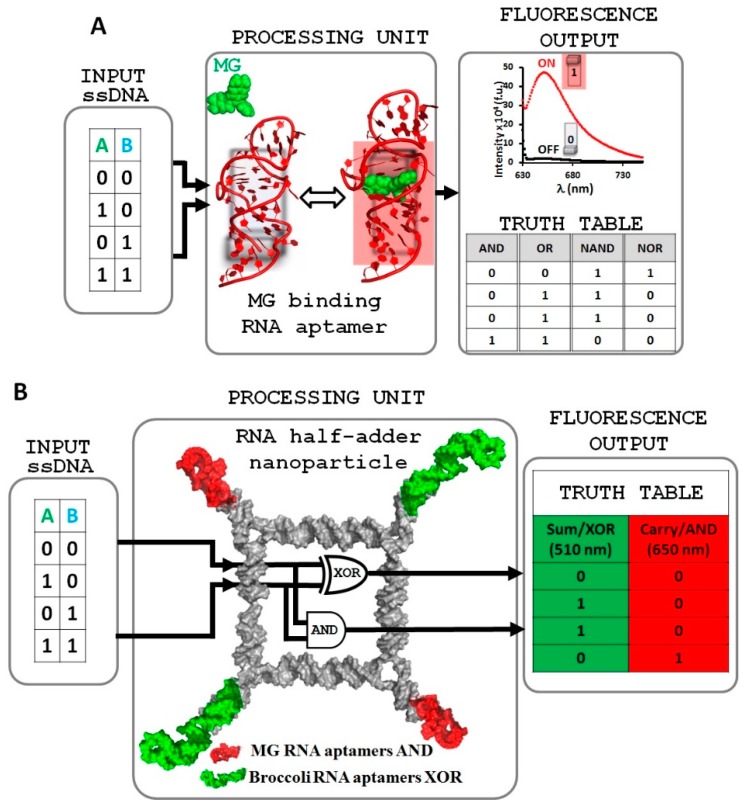
Logic gate design strategy based on light-up RNA aptamers. (**A**) malachite green (MG)-binding RNA aptamer used to design four simple AND, OR, NAND, and NOR logic gates. (**B**) Illustration of RNA half adder system based on MG RNA and Broccoli RNA aptamers conjugated with RNA tetragonal nanoparticle. Fluorophores MG and DFHBI in their unbound state exhibit low fluorescence (0), the emission of these chromophores increases upon binding to their corresponding RNA aptamers (1).

Herein, we report the design and assembly of a conceptually new generation of molecular logic gates that possess simple AND, OR, NAND, and NOR logic operations implementing the light-up MG-binding RNA aptamer. Single stranded DNA (ssDNA) oligonucleotides were used as inputs to trigger conformational changes in the RNA aptamer. The corresponding output values of OFF (0) and ON (1) are obtained by low and high fluorescence emissions, respectively (Figure 1A).

Furthermore, we developed a basic half adder computing platform based on the RNA light-up aptamer strategy (Figure 1B). The design utilizes fluorescence emissions from two distinct fluorogenic RNA modules, MG-binding RNA aptamer [27,59] and DFHBI-binding Broccoli RNA aptamer [29], fused with the previously reported tetragon RNA nanoparticle [8,9].

The function of the half adder is triggered by the same two ssDNA inputs. The ssDNA inputs alter the conformation of the RNA aptamers in such a manner as to either permit or deny fluorogen dye binding to the aptamer, resulting in fluorescent (ON) or non-fluorescent (OFF) states within one nanostructure. This RNA logic gate system demonstrates the great potential of light-up RNA aptamers as an arithmetic tool for molecular programming and will open a way to further development concerning well-regulated molecular electronic devices and biosensors.

## 2. Results and Discussion

### 2.1. Design and Fabrication of a Logic Gate Possessing AND, OR, NAND and NOR Boolean Functions

**Figure 2:**
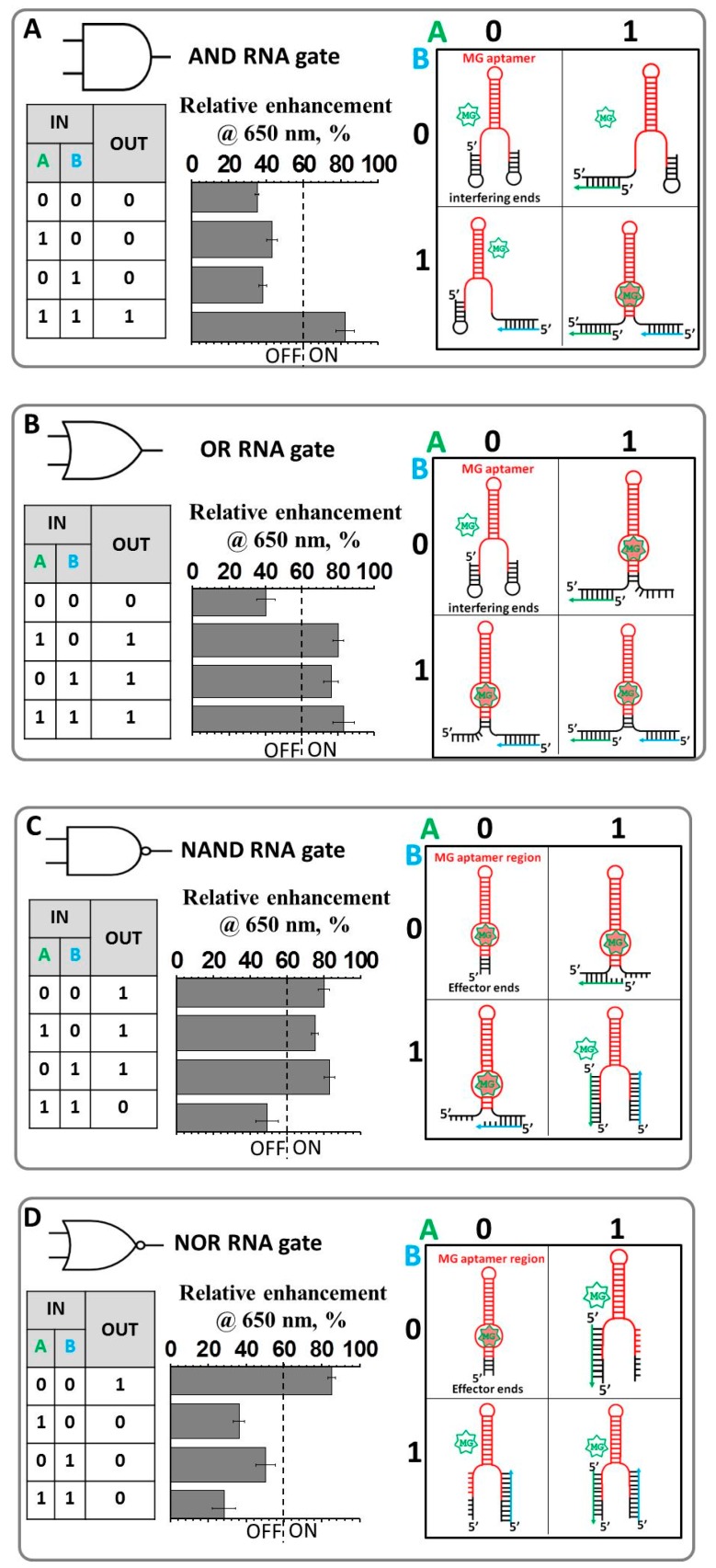
Logic gate design principles for an AND gate (**A**), OR gate (**B**), NAND gate (**C**), NOR gate (**D**). The predicted 2D conformations of the RNA aptamers in the presence and absence of individual or both inputs are shown to the right. The rules specified by each gate are shown in truth tables to the left. Normalized fluorescence enhancement of the gates is displayed in the middle. The fluorescence enhancement data are reported with ± standard error of the mean (SEM) bars; measurements have been reproduced from least three repetitive trials.

RNA molecules featuring aptamers that bind a fluorogenic dye and activate its fluorescence have the potential to be highly sensitive and convenient probes in the field of synthetic biology. The initial system is comprised of an RNA hairpin molecule containing the MG RNA binding aptamer sequence and the MG fluorogen dye [60]. The fluorescence of the MG dye is negligible when free in solution (OFF state) and is increased significantly upon binding to its RNA aptamer (ON state) (Figure 1). The principle of the LG design is based on the structural manipulation of the RNA aptamer, due to the fact that binding affinity of the fluorogenic dye to its light-up RNA aptamer depends on the correctly folded RNA structure. Thus, the fluorescence emission can be precisely turned ON (correctly folded RNA structure) and OFF (disrupted conformation). The four logic gates AND, OR, NAND, and NOR were designed using this highly effective and modular approach (Figure 2).

Individual gates employed light-up RNA aptamers consisting of two extended sequences localized at both the 5′- and 3′-ends highlighted in Figure 2 in black. This will further be referred to as the interfering end for the AND and OR gates or the non-interfering ends for the NAND and NOR gates. The MG RNA aptamer core sequence is highlighted in red. The end sequences are tailored to bind the ssDNA oligonucleotides which serve as inputs. All the RNA gates were designed in silico relying on secondary structure prediction algorithms encompassed within NUPAC [61] or mfold programs [62] to confirm the secondary structures of each RNA sequence prior to synthesis. If the calculated lowest free-energy secondary structure corresponded to the desired RNA conformation, and no other secondary structure was closer than 20% in energy to the lowest energy structure, the sequence was used without alternations. Otherwise, minor changes were made to Watson–Crick base-paired positions to destabilize competing conformations. The fluorescence was measured at 22 °C in 1× TMS buffer as described in the materials and methods. Each RNA logic gate was designed to have complementary regions at the 5′- and 3′- ends to ssDNA oligonucleotide inputs with lengths ranging from 15 to 27 nucleotides (Supplementary Table S1). Each gate processes a different pair of oligonucleotide inputs—for instance, input A of the AND gate is not the same as input A of the OR gate—and the terms A and B were merely applied across the table for simplicity. However, the sequences and therefore 2D structures for AND and OR gates were designed to be identical as both initial structures, at the no inputs setting, should have 0 output or exist in the OFF state according to the truth table (Figure 2A,B).

In a similar manner, initial structures for the NAND and NOR gates were chosen to share identical nucleotides as the ON state is a requirement for both structures at the no inputs condition. For each logic gate, the fluorescence intensities were normalized throughout the experiments. The threshold value was determined to be 60% where an intensity greater than this value yields an output = 1, while an intensity below this value yields an output = 0.

The AND and OR gates with default setting (0-0; no inputs present) was designed using interfering ends. The purpose of this was to form a complementary base pairing with the RNA aptamer MG-binding region as illustrated in Figure 2A,B. The interfering sequences at both ends were chosen to form weak hairpin-like 2D structures (2D structures available in Supplementary Figure S1). These hairpins are formed by involving core nucleotides that are responsible for forming a binding pocket for the MG fluorogen (Supplementary Figure S1). Thus, the presence of the hairpins prevents the light-up aptamer from binding to its MG chromogen and ultimately diminishing the fluorescent signal. The AND operation is achieved by carefully designing two short ssDNA inputs (A and B) to bind to the 5′ and the 3′ interfering ends, respectively. The structural rearrangement to the ON conformation occurs only when there are two inputs present at the same time. The presence of the inputs releases the core MG nucleotides (nts) thus allowing them to fold into the proper ON conformation. The selection of these particular ssDNA inputs was achieved by varying their length using RNA structure prediction programs (Supplementary Figure S2). The goal was to select the appropriate length of the inputs that would hybridize (or not hybridize at all) with its target hairpin without perturbing the conformation of the other hairpin when only one input is added. The selection of the DNA inputs was based on the computed melting temperature (T_m_), the temperature at which 50% of double stranded nucleic acid is converted to single-standard form. The desired Tm values for inputs hybridization were chosen to be slightly greater than 22 °C, the temperature at which the experiment was performed. Such input selection relies on the equilibrium between duplex (formed by ssDNA and hairpin nts) and hairpin structures where equilibrium shifts slightly in favor of the hairpin structure (Figure S2, AND GATE). However, inclusion of both inputs favors the formation of two duplexes triggering structural rearrangement of the overall complex, which leads to the liberation of the core nts accountable for MG-binding site formation. As shown in Figure 2A, resulting fluorescence intensity measured for four annealed samples (0-0; 0-1; 1-0, and 1-1) clearly indicates the effectiveness of the AND gate function.

The OR construct, which yields output = 0 for scenario 0-0 and output = 1 for scenarios 1-0, 0-1, and 1-1, shares the identical RNA sequence as the AND gate. However, the ssDNA inputs were designed to have several extra nucleotides to achieve an equilibrium state where inclusion of at least one input will trigger conformational change in favor of the correctly folded MG-binding RNA aptamer. The design criterion is based on a strand displacement reaction. Upon binding to its corresponding hairpin, input A or B will create a stable hybrid RNA/DNA duplex. The DNA inputs were selected to contain longer sequences with much higher Tm values as compared to the AND gate, so that disrupted hairpin nts initiate a conformational change of the whole construct favoring formation of the ON aptamer state (Figure S2, OR GATE). The measured fluorescence emission values in solution demonstrates OR behavior of the RNA construct (Figure 2B).

In contrast to the AND and OR gates, the NAND and NOR gates were designed so the default structures possess correctly folded light-up MG RNA aptamers and the extensions at the 5′- and 3′-ends do not interfere with the core structure. To produce the corresponding NAND operation, the non-interfering ends must be able to bind input A or input B without sacrificing the conformation of the aptamer. However, when both A and B are presented, the conformation of the aptamer needs to be sufficiently distorted to register an output = 0. Figure 2C summarizes the fluorescence enhancement measurement for the NAND gate. Interestingly, while input A alone (1-0) decreases fluorescence as compared to the 0-0 state, input B alone (0-1) increases it slightly. Inputs A and B in tandem (1-1) triggered a noticeable decrease in fluorescent intensity (Figure 2C).

The NOR logic gate was constructed utilizing the NAND RNA molecule. Figure 2D shows an obvious increase in fluorescence intensity between output = 0 and output = 1, owing to the nature of the RNA NAND logic gate. The designed ssDNA inputs that complimentarily pair with the RNA gate can significantly disrupt the conformation of the RNA molecule, rendering MG binding impossible. Hence, the output was “1” when only neither DNA input was added (Figure 2D).

Collectively, the modular approach to the fabrication of RNA Boolean logic gates based on the light-up RNA aptamer was demonstrated. All designed gates produced the expected OFF or ON values corresponding to low or high fluorescence intensity at λ_max_ = 650 nm, respectively, in response to DNA oligonucleotide inputs. A threshold value of fluorescence enhancement of 60% was chosen to distinguish the OFF (any value bellow 60%) and ON (any value above 60%) states. Various concentrations of RNA molecular gates, inputs, and MG dye in solution were explored, with those yielding the greatest difference in fluorescence between output = 0 and output = 1 reported here. The extent to which these modular RNA logic gates can be used to probe three or more inputs simultaneously will depend on their reliability in tandem.

### 2.2. Implementing Logic Gates to Construct a Half-Adder Logic Circuit

**Figure 3:**
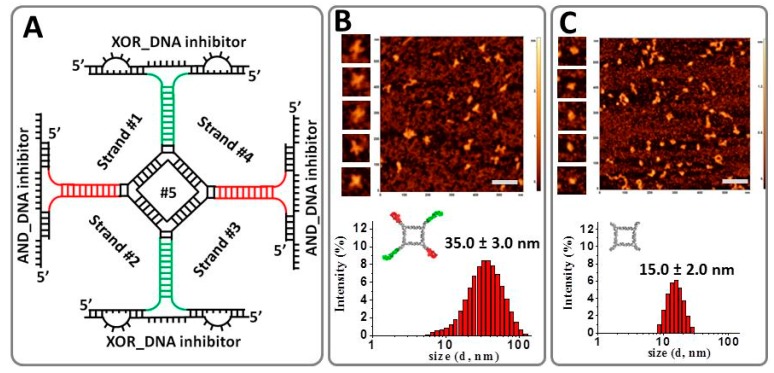
Implementation principle of the developed half adder. (**A**) Secondary structure of the RNA complex based on MG and Broccoli RNA aptamer conformations fused to tetragonal nano-scaffold. (**B**) Representative atomic force microscopy image (AFM) of the RNA half adder (bar scale = 100 nm) and Dynamic Light Scattering (DLS) data showing average size of the complex ± SEM. (**C**) AFM and DLS data for control RNA tetragonal nanoparticle, with the diameter of the nanoparticles is reported.

The production of multi-functional nanodevices for data analysis or processing is extremely important and yet challenging due to requirements of multiple coordinated logic gates operations within a single unit. Based on the aforementioned results, we next integrated two different fluorogen-binding RNA aptamers: (i) MG RNA aptamer and (ii) the recently developed Broccoli RNA aptamer that binds DFHBI dye within one RNA complex. As a key building block, the half adder is used to construct more advanced computational circuits and is in high demand in information technology [63]. The representative secondary structure of this complex is demonstrated in Figure 3A. The differences in the emission properties of these two fluorogens (MG emits in the “red” region while DFHBI emits in the “green” region of the visible spectra range) were implemented to construct a half-adder logic circuit, which is a primary step in constructing a full adder, a basic arithmetic unit in computing.

**Figure 4:**
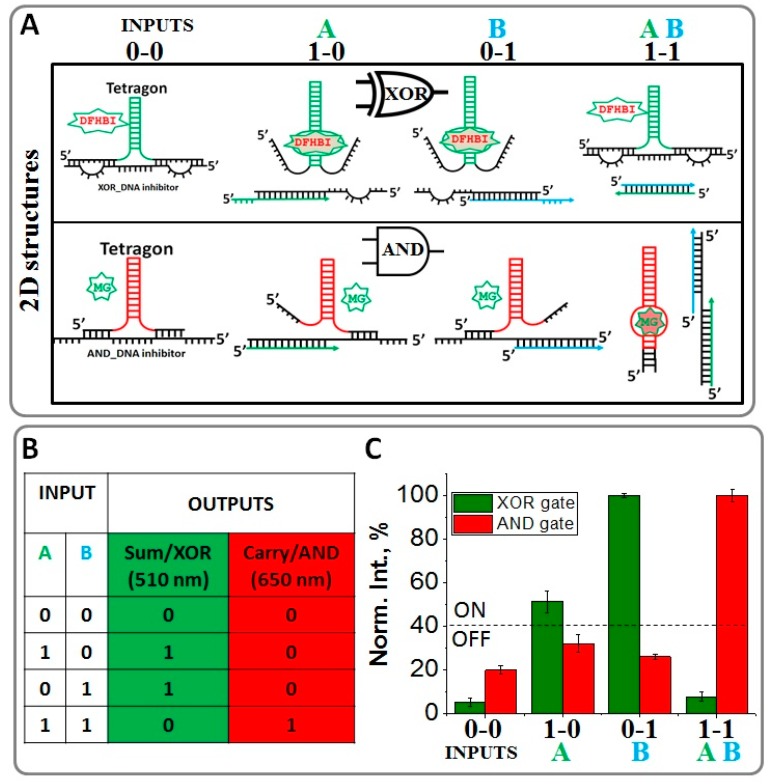
Design principles of the RNA AND and XOR gates using inhibitor DNA strand. (**A**) Predicted secondary structures of the nucleic acid displacement reactions within XOR and AND gates. (**B**) Truth table of a half adder. (**C**) The normalized fluorescence enhancement of the system at 510 nm and 650 nm as a function of the various inputs (Inputs A and B); the error bars indicate ± SEM from three independent measurements.

The half adder is composed of an AND gate with a MG-binding light-up RNA aptamer, and a XOR (eXclusive OR) gate based on the DFHBI-binding Broccoli light-up RNA aptamer [29]. The fluorescent intensity was measured in solution using fluorescent spectroscopy with excitation wavelengths of λ_ex/em_ = 465/510 nm (corresponding to the XOR gate) and λ_ex/em_ = 615/650 nm (corresponding to the AND gate). These gates were rationally designed to use two ssDNA inputs to output two fluorescence signals: SUM (λ_em_ = 510 nm) and CARRY (λ_em_ = 650 nm) generated by the AND and XOR gates, respectively (Figure 4). Both the MG RNA aptamer and Broccoli RNA aptamers were incorporated on alternating vertices to a previously developed RNA tetragon nanoparticle. The RNA half adder self-assembles from five RNA strands with a yield exceeding 80% (Supplementary Figures S3 and S4). The conformation of the assembled RNA tetragonal geometry was confirmed by atomic force microscopy and size was determined by dynamic light scattering (Figure 3B). Atomic Force Microscopy image (AFM) imagining revealed the extensions at each vertex in the designed RNA half adder as compared to the control RNA square nanoparticle. The size of the nanoparticle increases from 15 nm to approximately 35 nm with the addition of the RNA aptamers as shown by Dynamic Light Scattering (DLS) experiment. Also, the significant size variation between RNA tetragon and RNA half-adder nanoparticles was confirmed by native polyacrylamide gel electrophoresis (PAGE) (Figures S5 and S6).

To perform the XOR and AND logic operations using the same two ssDNA inputs, additional DNA inhibitor strands were introduced to bind complementary to the light-up RNA aptamers and interfere with their ON states or correctly folded structures. This tetragonal shaped half-adder RNA complex is designed according to the competitive hybridization and displacement principle of the DNA strands. The assembly experiments shown in Figure S5 confirm complexation of both inhibitors with the tetragonal nanoparticle. Importantly, the RNA tetragon containing 2 MG and 2 Broccoli RNA aptamers assembles with their corresponding DNA inhibitors at 1:2 ratio, i.e., one tetragon and 2 AND_DNA inhibitors and 2 XOR_DNA inhibitors.

The Broccoli RNA aptamer was designed to act as an XOR gate. To maintain the proper XOR gate function, an inhibitor DNA strand was designed (XOR_DNA inhibitor). The inhibitor bound to the aptamer, disrupting the binding of DFHBI and thus diminishing fluorescence in the presence of neither input. This XOR_DNA inhibitor contains two loop regions on either side of the aptamer. These internal loops contain eight unpaired nts and are designed to complement the ssDNA inputs. The addition of either ssDNA input destabilizes the RNA aptamer-inhibitor complex, separating the strands enough to reform the functional RNA aptamer shape allowing (1-0) or (0-1) truth values in fluorescence with an output of “1” (Figure 4A).

However, in the presence of both inputs, fluorescence is once again inhibited as the hybridization of inputs A and B is favored over hybridization with XOR_DNA inhibitor. To achieve this, inputs A and B were designed to bind more competitively to one another than to the XOR_DNA inhibitor through 17 “sticky” nts at the 5′ end of input A and 3′ end of input B (Table S1). Therefore, the XOR_DNA inhibitor paired with the RNA aptamer yielded a low fluorescent output signal. Figure 4C shows the normalized fluorescence intensity of the designed XOR system at 510 nm output readout in response to the ssDNA inputs. The presence of each input is defined as “1” (the absence is considered “0”) and the output signal is defined as ON or OFF when the normalized fluorescence emission is higher or lower than 40%, respectively. The system exhibits ON in the presence of the individual inputs; otherwise, it remains OFF. The XOR logic operation performs the SUM digit function in the half adder as shown in the Truth Table (Figure 4B).

The AND logic operation of the half adder was designed utilizing the fluorescent properties of the MG-binding RNA aptamer system as the output signal. Similar to the XOR gate approach, a DNA inhibitor (AND_DNA) was used to disrupt the RNA aptamer conformation. The AND gate has an output of OFF or (0) in the absence of inputs (0-0) or in the presence of only one of the inputs (1-0 or 0-1). As the inputs are the same as those used for the Broccoli XOR gate, it is critical for the AND_DNA inhibitor to be complementary to the previously designed inputs. For this purpose, the AND_DNA inhibitor was designed to contain “sticky” nts at the 5′- and 3′-ends. These “sticky” nts are complementary to the ssDNA inputs. The addition of either input causes only a partial displacement of the AND_DNA inhibitor from the light-up RNA aptamer resulting in the low output value as demonstrated in Figure 4C. However, in the presence of both inputs, fluorescence increases significantly (ON state). This was accomplished by disassociating the AND_DNA inhibitor. The ssDNA inputs bind more competitively to the inhibitor to form a three-stranded DNA/DNA/RNA complex enabling the successful separation from the RNA light-up aptamer. Figure 4A (lower panel) summarizes the 2D structures computed for the AND logic system in the presence and absence of the ssDNA inputs. The normalized fluorescence intensities of the system at 650 nm as a function of the inputs are plotted in Figure 4C indicating that the system exhibits “1” only when both inputs coexist, indicative of an AND logic gate. The AND logic gate is responsible for the CARRY digit function in the half adder, as shown in the truth table in Figure 4B. To conclude, the AND and the XOR gates were implemented in parallel utilizing light-up RNA aptamers as a label-free fluorogenic platform. Both gates were triggered by the same set of inputs, satisfying the requirements for a half adder [64]. The further development of the full adder system based on the RNA high-up aptamers is currently under investigation. By definition, the full adder should perform an addition operation on three binary digits and similarly to the half adder, it generates a carry out to the next addition column. This development requires three inputs, which can be the same two ssDNA inputs and an additional carry-in DNA input to receive the carry signal from a previous stage.

## 3. Materials and Methods

### 3.1. Nucleic Acid Sequence Design, Synthesis, and Assembly

Polynucleotide sequence design was carried out using the multi-strand secondary structure prediction programs NUPACK and mfold [61,62]. To meet the requirements of the developed logic gates, the DNA and RNA sequences used in the experiments were first designed and then analyzed by the above 2D structure folding predicting software. According to the predicted 2D structures, experiments were performed to determine whether the designed ssDNA oligonucleotides were operational in the corresponding logic gate processing reactions. If the satisfying fluorescence readouts were not achieved, the DNA sequences were redesigned and the procedures were repeated until the desired DNA sequences obtained.

All DNA oligonucleotides were purchased from IDT DNA (Coralville, IA, USA) as desalted products and used without purification. RNA strands corresponding to individual logic gates and to the tetragonal half-adder complex were prepared by in vitro transcription using T7 RNA polymerase [8]. For this, synthetic DNA strands coding for the anti-sense sequence of the RNA strands were amplified by polymerase chain reaction (PCR) using primers containing the T7 RNA polymerase promoter. PCR products were purified using the QiaQuick PCR purification kit (Qiagen Sciences, Germantown, MD, USA). The transcribed RNA molecules were purified by denaturing 20% polyacrylamide gel electrophoresis containing 8M UREA.

The self-assembly of individual MG-based light-up RNA logic gate complexes AND, OR, NAND, and NOR was achieved by mixing equimolar oligonucleotide strands (1 μM) in TMS (50 mM TRIS pH = 8.0, 100 mM NaCl and 10 mM MgCl_2_) buffer and heating the mixture to +80 °C and gradually cooling it down to +4 °C over a period of 1 h on a PCR thermocycler. Once the RNA aptamer self-assembly was achieved, a small amount (2 μM final concentrations) of the malachite green oxalate salt (Sigma Aldrich Co., St. Louis, MO, USA) was added to each RNA or RNA/DNA assembly. The mixture was left to incubate for an additional 30 min at 22 °C to reach proper binding equilibrium. DNA inputs oligonucleotides were added to the assembled AND, OR, and NOR gates at the final concentrations of 2 μM (each) making the final stoichiometry of the complexes as follows:

1 GATE: 1 INPUT A: 1 INPUT B.

For the NAND system the optimal results achieved at stoichiometry:

1 NAND: 2 INPUT A: 2 INPUT B.

The self-assembly of the half-adder RNA construct was achieved by mixing corresponding RNA and DNA polynucleotides at 1:1 stoichiometric ratio. For example, the RNA half adder in the absence of inputs contained 1 μM of each RNA strands, 2 μM of AND_DNA inhibitor, and 2 μM XOR_DNA inhibitor. Malachite green and 3,5-difluoro-4-hydroxybenzylidene imidazolinone (DFHBI) (Sigma Aldrich Co., St. Louis, MO, USA) dyes were added to the corresponding complexes (0-0, 0-1, 1-0, 1-1) to make 5 μM final concentration. The resulting mixture was allowed to incubate for an additional 30 min at 22 °C. After reaching equilibrium, DNA inputs (5 μM each) were added in accordance to the truth table for the half-added RNA systems and fluorescence were recorded after additional 30 min incubation, which was necessary to achieve input driven strand displacement effect (Figure S8)

### 3.2. Fluorescence Measurements

Fluorescence was measured on a Fluoromax-3 (Hibora Jobin-Yvon, Horiba Scientific, Edison, NJ, USA) spectrofluorimeter using a Sub-Micro quartz fluorometer cell (Starna cells Inc., Atascadero, CA, USA). Fluorescence intensities were recorded separately for each dye. For the DFHBI-binding Broccoli RNA aptamer, the excitation wavelength centered at 465 nm and emission was collected in the range of 475–700 nm. For the MG-binding RNA aptamer, the excitation was centered at 615 nm and emission was recorded from the range of 630–750 nm.

The fluorescent enhancement was quantified by the ratio of the maximum emission of the fluorogenic dyes bound to its aptamers divided by the emission of the free dyes in solution. The fluorescence enhancement data were normalized after the experiments; a threshold value was chosen to be 60% for the MG based RNA logic gates, an intensity greater than this value yields an output = 1, while an intensity below this value yields an output = 0.

### 3.3. Dynamic Light Scattering

Hydrodynamic diameters of assembled half-adder RNA constructs and the control tetragon RNA nanoparticles were measured by a Zetasizer nano-ZS (Malvern Instrument Ltd., Malvern Panalytical Ltd., Malvern, UK) at 22 °C following previously described protocols [9].

### 3.4. Atomic Force Microscopy Imaging

The RNA tetragon and RNA half-adder complexes were imaged with MultiMode AFM NanoScope IV system (Veeco Instruments Inc., Plainview, NY, USA), following previous methods [65].

## 4. Conclusions

Molecular logic gates hold great potential for a wide range of biotechnological applications, including gene expression regulation, biosensors, therapeutic molecule design, metabolic reprogramming, studies of drug-nucleic acid interactions, and tools for elucidating cellular functions. The emergence of RNA nanotechnology offers great opportunities for applications of RNA-based logic gates. In this study, we have used a computational approach to design various oligonucleotide-responsive RNA logic gates (AND, OR, NAND and NOR) based on the MG-binding RNA aptamer. The structures of four logic gates were designed based on the general 2D architecture depicted in Figure 2 and all functioned as robust RNA switches that exhibit fluorescence emission once activated. The design process used here accounts for the thermodynamic stability of various base-paired structures in the absence or presence of input oligonucleotides. This functional design was possible due to the fact that nucleic acid secondary structure folding largely follows the simple rules of Watson-Crick base pairing, and the thermodynamic parameters for base-pair interactions are available. In addition, a half adder was successfully demonstrated by combining the hybridization and replacement of ssDNA strands. Specifically, introducing two light-up RNA aptamers MG and Broccoli into a half-adder system to modulate the output signal makes it flexible and enables the potential design of various other types of logic gates according to the requirements of the data processing. Although the developed individual logic gates and half adder are implemented in an experimental stage and exclusively in vitro, the demonstrated system presents great potential for the development of other RNA-light up based logic circuits as a universal arithmetic tool. To summarize, this work provides a novel light-up RNA aptamer-based platform for the design and assembly of higher-order circuits for arithmetic operations and opens the possibility to develop a new approach for constructing multicomponent devices on a single biomolecular nano-platform.

## Supplementary Materials

The following are available online at http://www.mdpi.com/2079-4991/8/12/984/s1.
