# Supplementary material for: Fluorogenic RNA Aptamers: A Nano-platform for Fabrication of Simple and Combinatorial Logic Gates
Source: Nanomaterials (Basel). 2018 Nov 28;8(12):984. doi: 10.3390/nano8120984 (PMC6315349; doi:10.3390/nano8120984)

Supporting Information

Fluorogenic RNA Aptamers: a Nano-platform for Fabrication of Simple and Combinatorial Logic Gates.

Victoria Goldsworthy, Geneva LaForce, Seth Abels and Emil F. Khisamutdinov*

Department of Chemistry, Ball State University, Muncie, IN 47304, USA

*Address correspondence to:
Emil F. Khisamutdinov, phone 765-285-8084; email: kemil@bsu.edu

Table 1. List of DNA and RNA sequences used to create logic gates and half adder.

Name	Sequence 5'- 3'
RNA AND/OR gate	gguuucaaccuuugcaaacaugcuuu**GGAUGGUAACGAAUGACCAGCCCGAAAGGGCUGGUCCG ACAUCC**uuugggaaauccuuugggugccguuu*
DNA AND_input A<br>DNA AND_input B	GTAAGCTTTGGTTGA (15 nt)<br>AAACAGCGTCCAGAGGAT (18 nt)
DNA OR_input A<br>DNA OR_input B	GCATGTTTGCAAAGGTTGAAA (21 nt)<br>AAACGGCGTCCAAAGGATTTCCC (22 nt)
RNA NAND/NOR gate	gguuugcaaacaugcuuu**GGAUGGUAACGAAUGACCAGCCCGAAAGGGCUGGUCCG ACAUCC**uuugggaaauccuuu
DNA NAND_input A<br>DNA NAND input B	CTATAGCATGTTTGC (15 NT)<br>**TTGATTTCCCTTTGGT** (16 NT)
DNA NOR_input A<br>DNA NOR_input B	CCATCCAAAGCATGTTTGCAAA (22NT)<br>AAAGGATTTCCCAAAGGATGTCGGACC (27 NT)
RNA half adder Strand #1	GGUGCCCGCUGACCGGAGACGGUCAAAUAGAGCAC**A**GGAUGGUAACGAAUGACCA GCCCGAGGUCGCGACCUUCUUUUCCCUCGCGACUCGAAAUGUUUCUUUUCGAGGUCG CCCCG**UAUCUGUCGAGUAGAGUGUGGGCUCGCACGCGGCUGCC** (155 nt)
RNA half adder Strand #2	**GGCGGCTGCCGCGCAGAGACGGUCGGGUCCAGAUA**UUGGAUCUUUCGCCUUUUCG CGAUACGGGCCAACGAUGGGUUUUGAAGGUCGCGACAAGGGCUGGTCCGACAUCCCA CA CAAATAGAGT GTGGGCCGAG CAGCGGCACC (144 nt)
RNA half adder strand #3	GGUGCCCGCUGACCGGAGACGGUCAAAUAGAGCACAGGAUGGUAA**C**GAAUGACCA GCCCGAGGGCGACCUCGUUUUGUACCAGCAUCCUCUUCAUAAGUUUUGGCGAAAGAU CC**CGUAUCUGUCGAGUAGAGUGUGGGCUCGCACGCGGCUGCC** (155 nt)
RNA half adder strand #4	**GGCGGCUGCCGCGCAGAGACGGUCGGGUCCAGAUA**UUGGGCGACCUCGUUUUCCG GGAUAUGGUCUCGGCCAACUUUUCGAGGUCGCCCAAGGGCUGGUCCGACAUCCCACA CAAAUAGAGUGUGGGCCGAGCAGCGGCAGC (142 nt)
RNA half adder strand #5	GGAUGCUGGUACUUUUGUUGGCCGAGACCAUAUCCCGGUUUUGAAACAUUUCGAGUC GCGAGGGUUUUCCCAUCGUUGGCCCGUAUCGCGUUUUCUUAUGAAGA (103 nt)
AND_DNA Inhibitor	ATAACAGCGCGTAGGGCCACGCTGCCGCTGCTCGGCCCACACTCTATTTGACCG GGATGTCGGACCAGCTGGTCATTCCTTACCATCCCACACTCTATTTGACCGTCTCC GGTCAGCGGGCAGGCGAGCCCTACGCGCTGTTAT (144 NT)
XOR_ DNA Inhibitor	GGCAGCCGCGTGTGCCGCTGGCTCGGCCACACTCTATTTGACCGTCCGGTGCGAGCTGC GCGGCGAGCCCTA (72 NT)
Input A_5' INPUT	ATAACAGCGCGTAGGGCTCGCCGGCCCGCTGACCGGAGACGGTCAAATAGAGTGTG (56 NT)
Input B_3' INPUT	CGGTCAAATAGAGTGTGGGCCGAGCAGCGGCAGCGTGGCCCTACGCGCTGTTAT (54 NT)

* Lower letters indicate interfering (AND/OR Logic Gates) and non interfering (NAND/NOR logic gates) nucleotides. RNA nucleotides forming MG structural motif colored in red. RNA nucleotides that correspond to the Broccoli aptamer are in green color.

Figure S1. Secondary structures of designed AND/OR (left) and NAND/NOR (right) gates predicted by *mfold* and NUPAC. The 2D structure of the MG aptamer region is highlighted by the red colored nucleotides and by a rectangle.

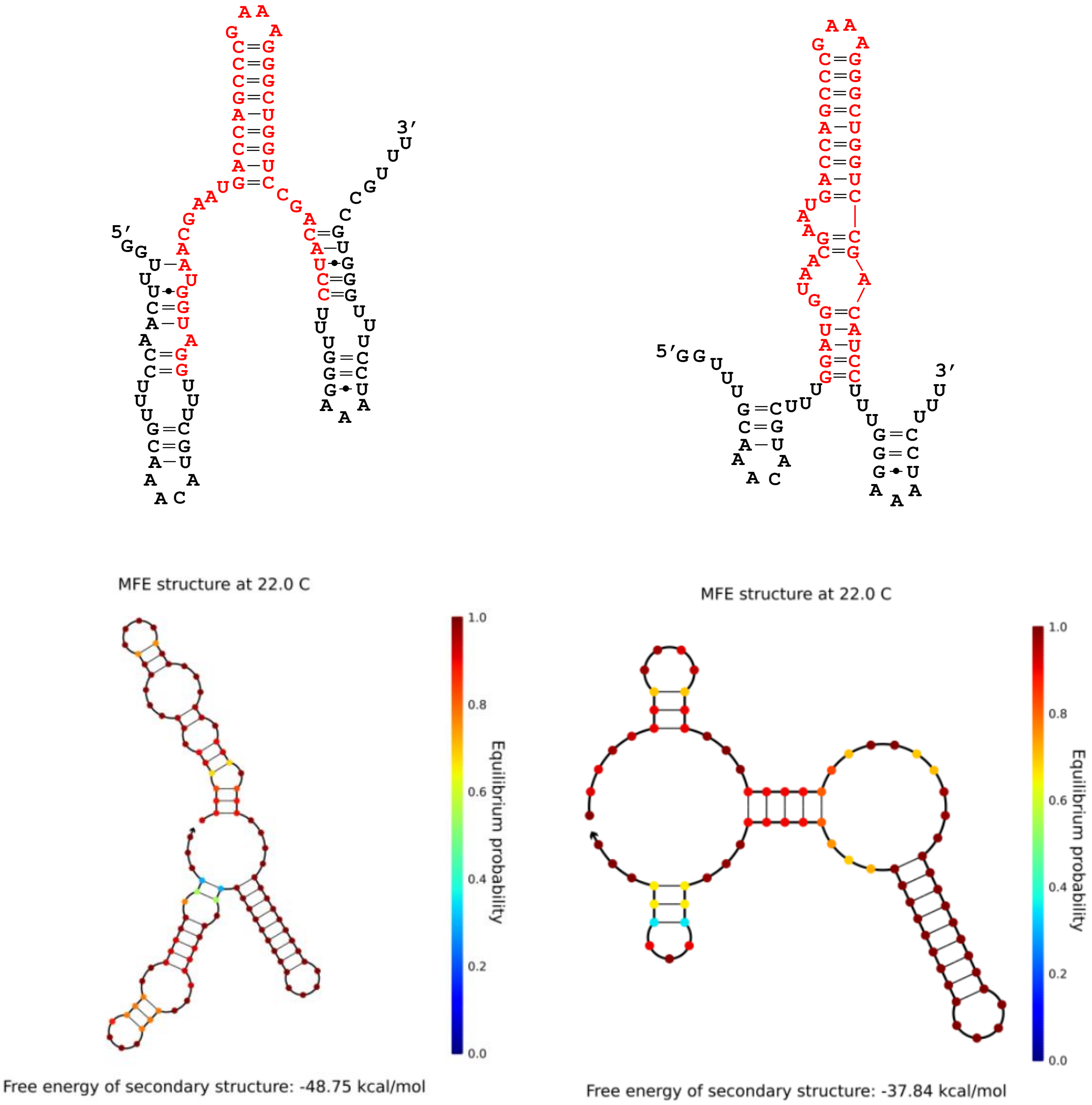

Figure S2. Predicted secondary structures of designed AND and OR gates complexes with and without inputs and their corresponding fluorescence intensities.

AND GATE

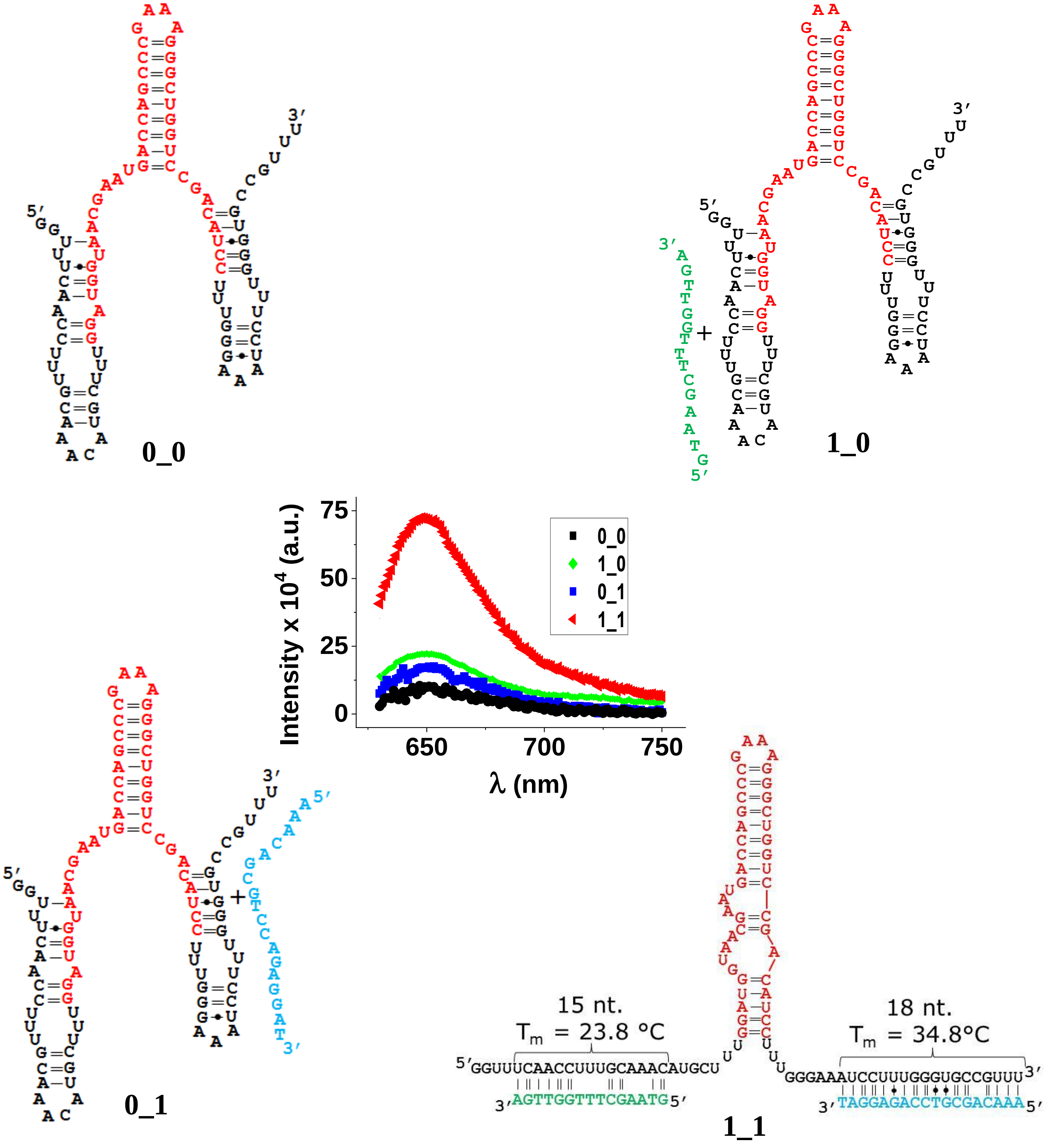

OR GATE

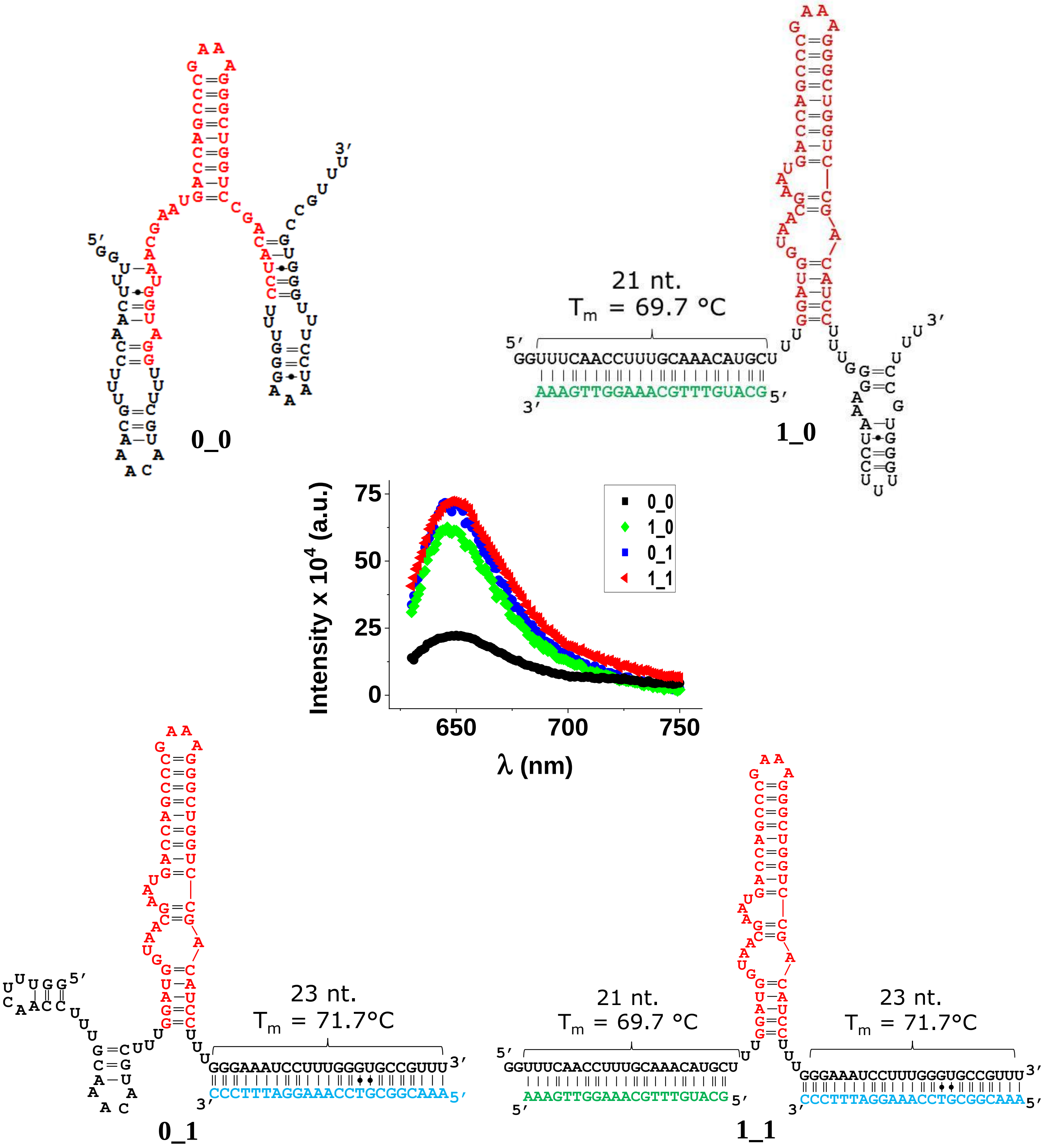

0_0
1_0
0_1
1_1
21 nt.
T_m = 69.7 °C
23 nt.
T_m = 71.7°C
5′ GGUUUCAACCUUUGCAAACAUGCU
3′ AAAGTTGGAAACGTTTGUACG 5′
GGGAAAUCCUUUGGGUGCCGUUU 3′
3′ CCCTTTAGGAAACCTGCGGCAAA 5′
Intensity x 10^4 (a.u.)
λ (nm)
0
25
50
75
650
700
750
0_0
1_0
0_1
1_1

NAND GATE

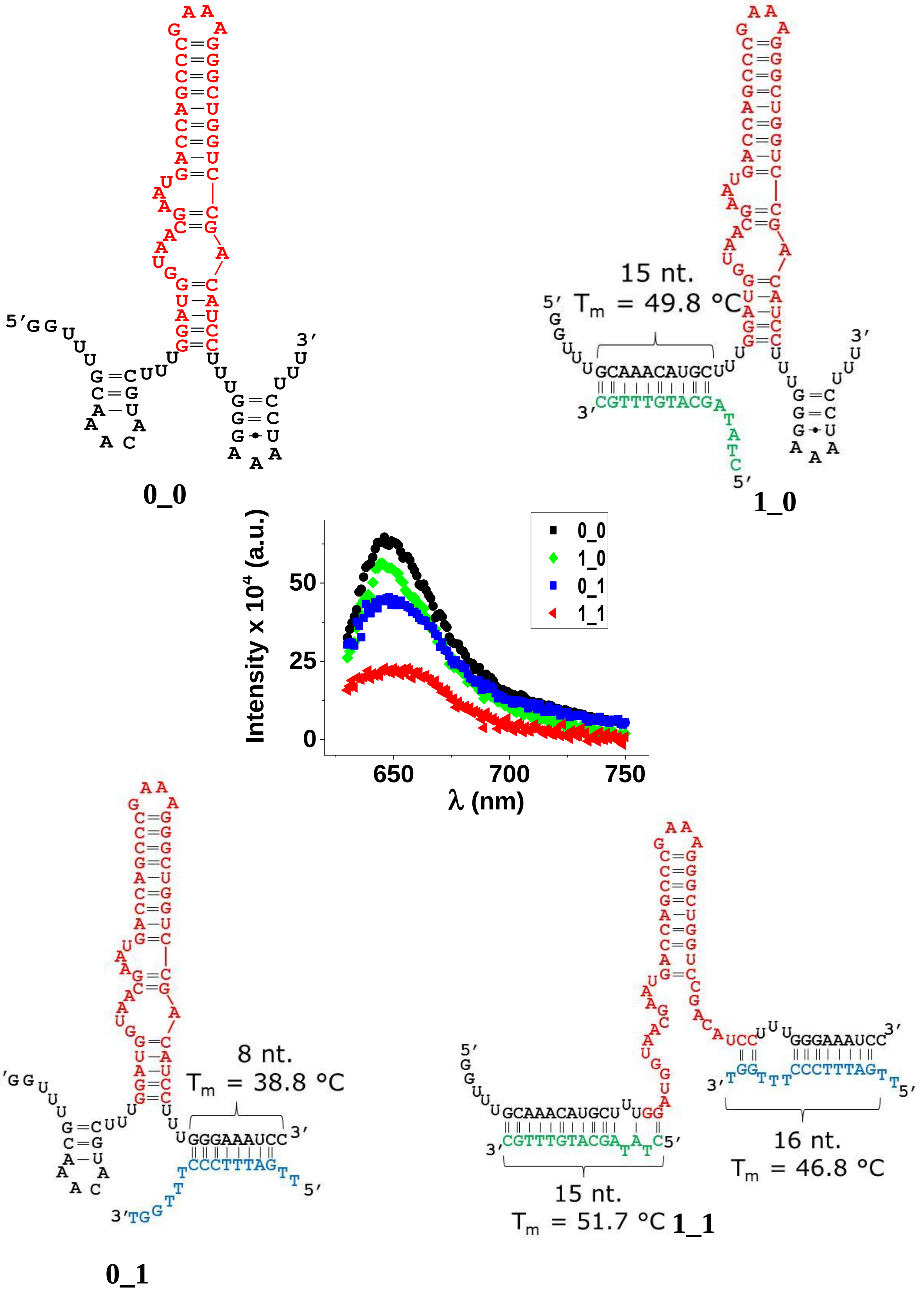

0_0
1_0
0_1
1_1
15 nt.
T_m = 49.8 °C
8 nt.
T_m = 38.8 °C
15 nt.
T_m = 51.7 °C
16 nt.
T_m = 46.8 °C
GCAAACAUGCU
CGTTTGTACGA
GGGAAAUCC
CCCTTTAG
Intensity x 10^4 (a.u.)
λ (nm)
0_0
1_0
0_1
1_1

NOR GATE

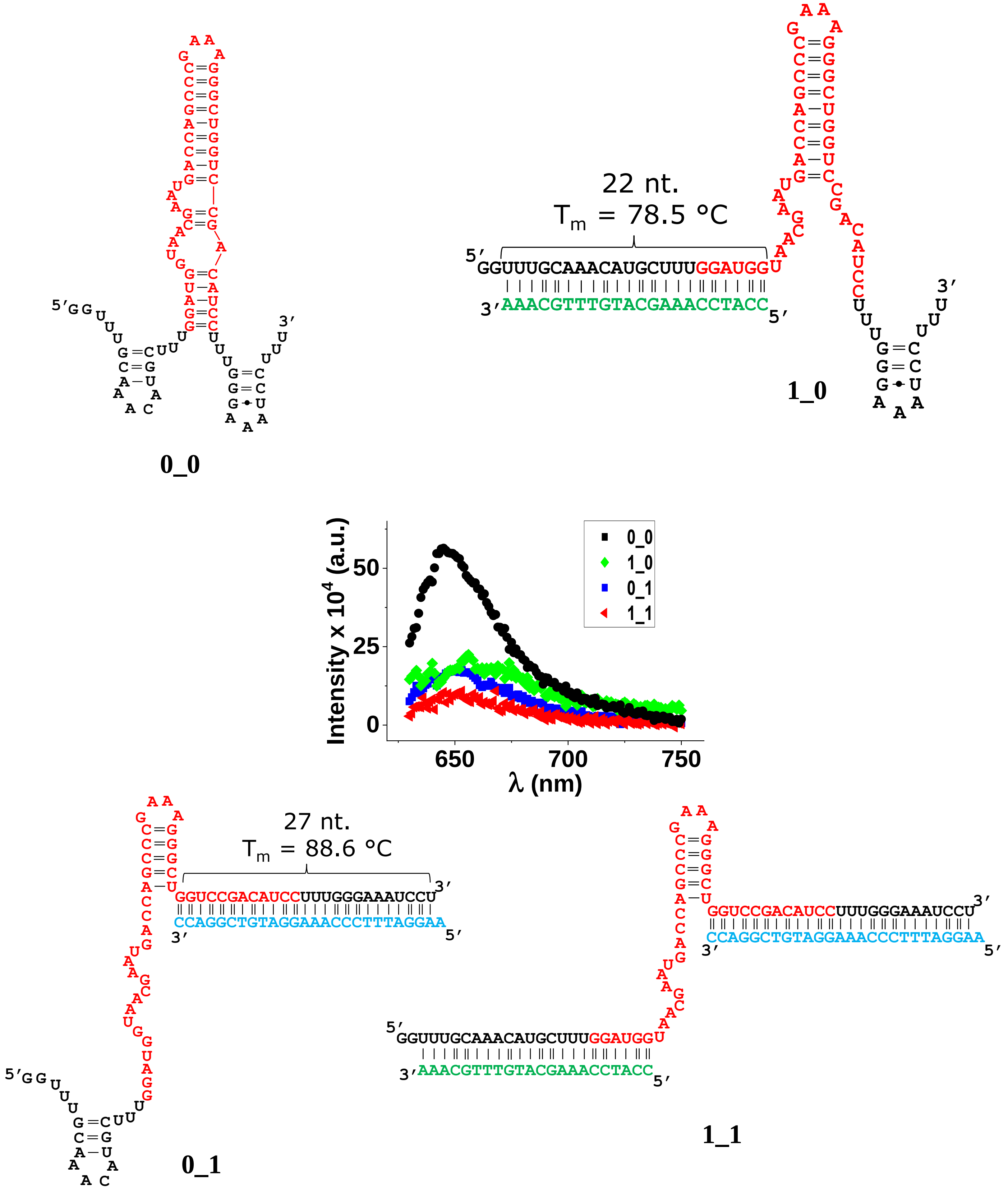

0_0
1_0
0_1
1_1
22 nt.
Tm = 78.5 °C
5′ GGUUUGCAAACAUGCUUUGGAUGGU
3′ AAACGTTTGTACGAAACCTACC 5′
27 nt.
Tm = 88.6 °C
GGUCCGACAUCCUUUGGGAAAUCCU 3′
3′ CCAGGCTGTAGGAAACCCTTTAGGAA 5′
Intensity x 10^4 (a.u.)
λ (nm)
0
25
50
650
700
750

Figure S3. Assembly assay of RNA half adder with no DNA inhibitor strands evaluated by 3% agarose gel. Lane L refers to DNA ladder (Low Molecular Weight, New England Biolabs), representative 2D structure of the RNA complex is shown on the left.

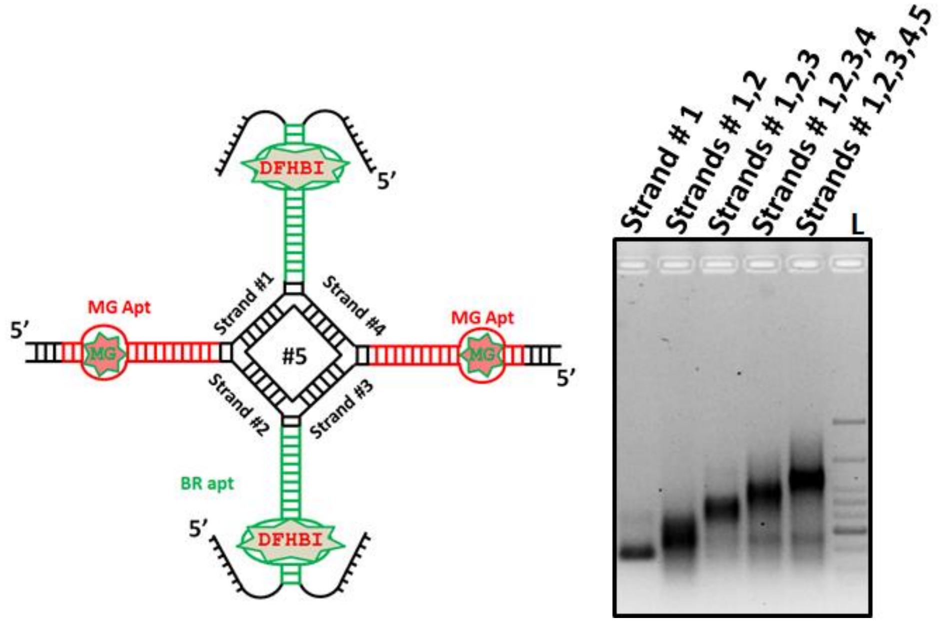

Figure S4. Secondary structure and sequences of designed Half-Adder RNA system with no Inhibitors present.

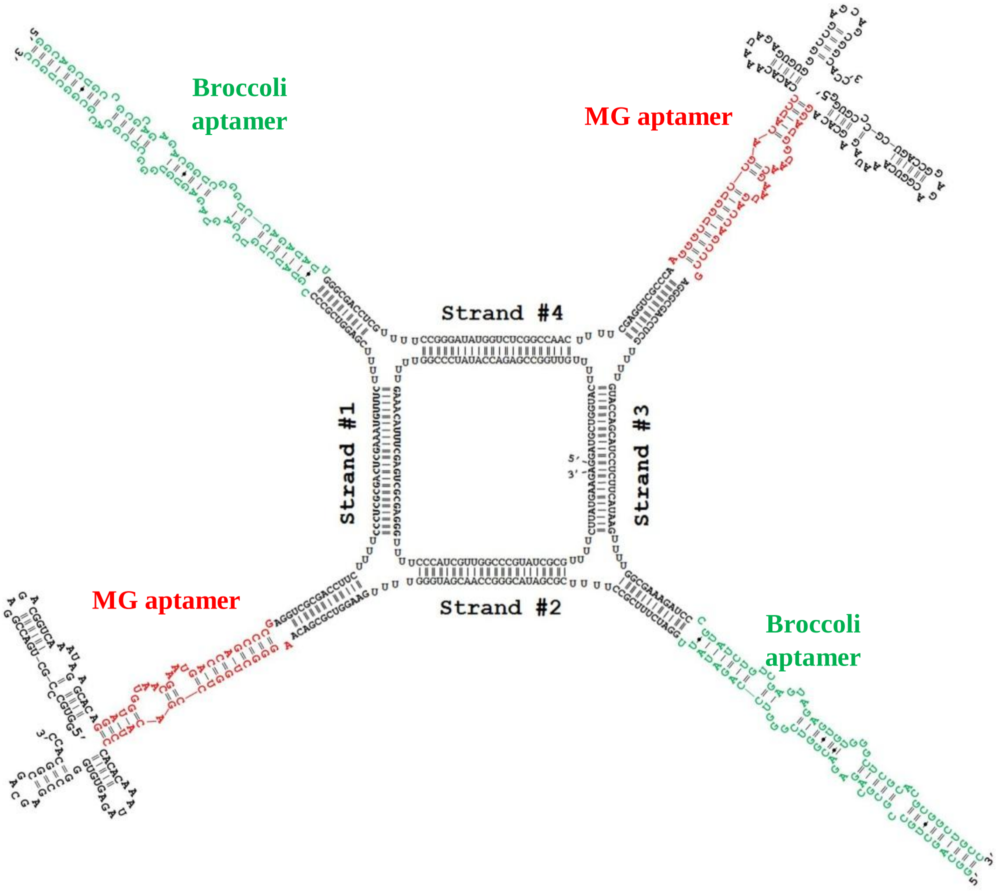

Figure S5. Comparison of electrophoretic mobilities of RNA half adder and RNA tetragon. Evaluation performed on native 7% gel. Lane L refers to DNA ladder (Low Molecular Weight, New England Biolabs).

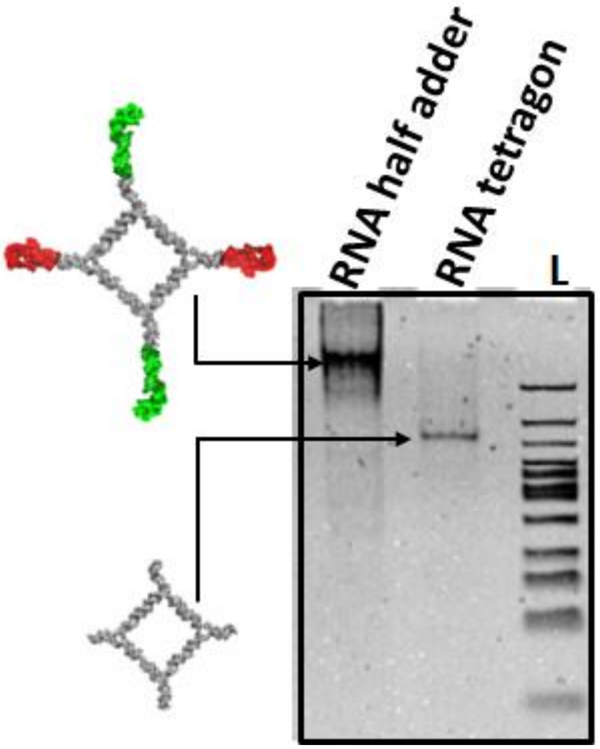

Figure S6. Assembly assay of RNA half adder with increasing concentration of DNA XOR and AND inhibitors mixture evaluated by 3% agarose gel. Lane L refers to DNA ladder (Low Molecular Weight, New England Biolabs).

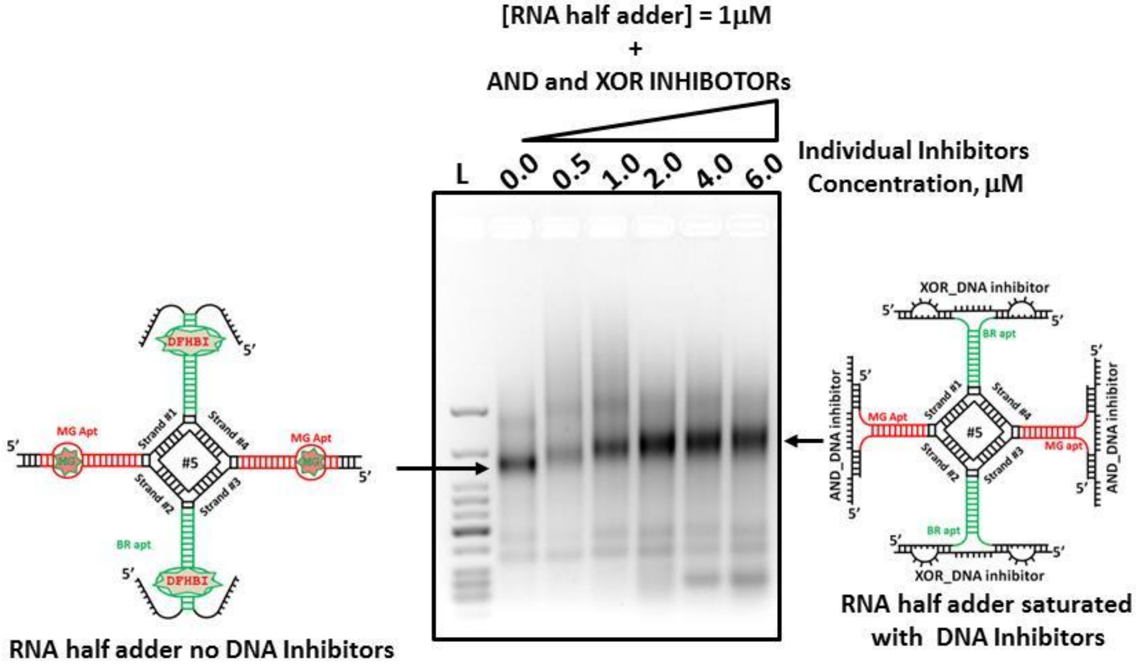

Figure S7. Computed 2D structures of the designed half adder containing XOR and AND logic gates with their inhibitors as a function of either input or presence of both. For the simplicity, the structure of the tetragon is shown with either (A) Broccoli aptamer or (B) MG RNA aptamer. Highlighted by the green and red boxes are the correctly folded 2D structures of Broccoli aptamer and MG aptamers, respectively. The structures were obtained from NUPAC program.

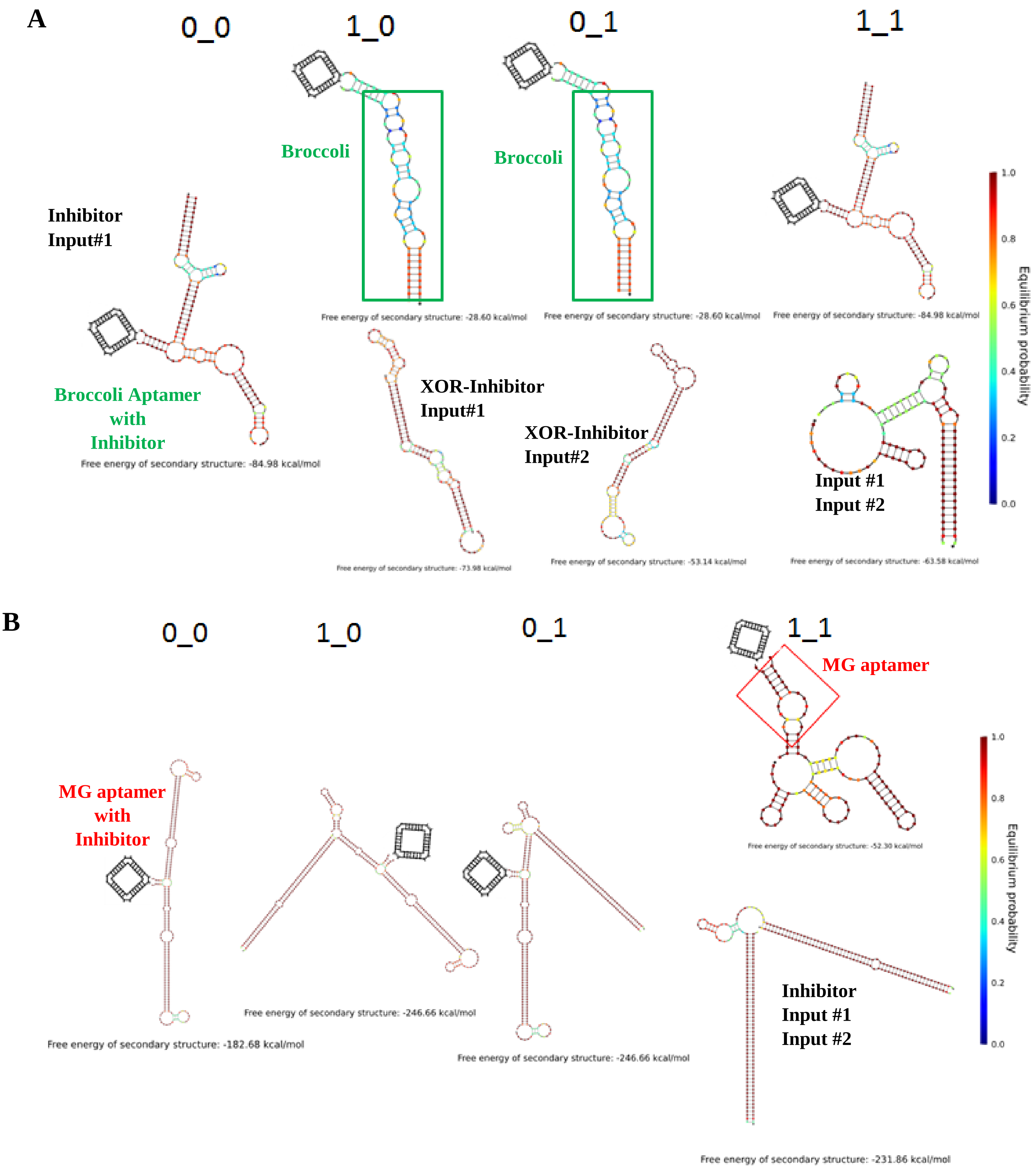

Figure S8. Time dependent fluorescence intensity of the AND gate (RNA MG aptamer) of the half adder in presence of two DNA inputs. To the preassembled tetragonal half added (1 μM) containing XOR and AND inhibitors (2 μM) a small volume of both DNA inputs A and B were added to achieve 5 μM final concentration of each. The fluorescence measured at different time intervals at 22 ℃.

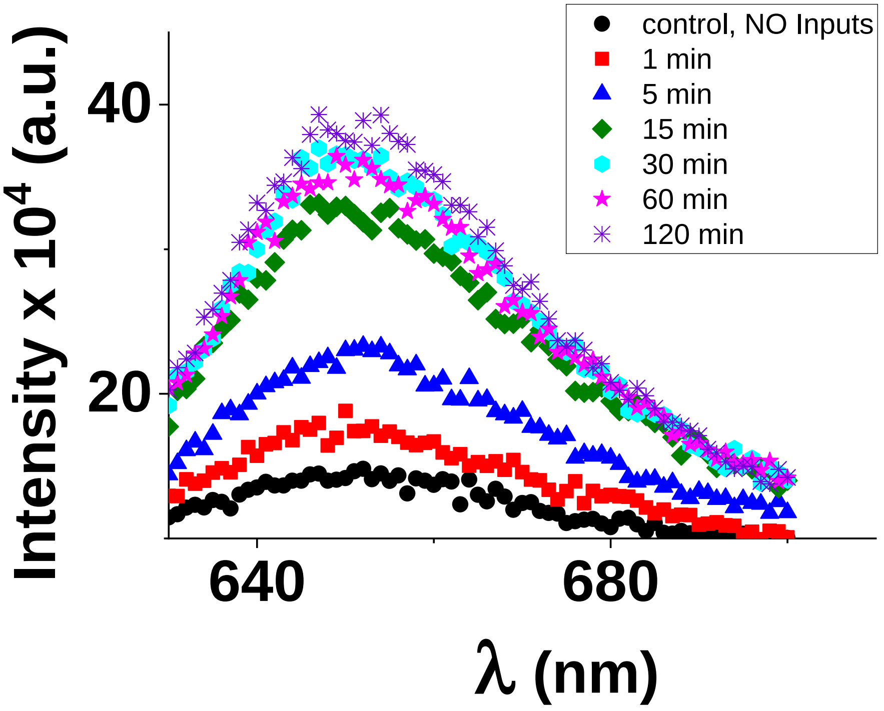

Supplement: Supplementary file 1 [file nanomaterials-08-00984-s001.pdf]
